# Development of A Nomogram for Progression-free Survival in Patients with Stage II/T3N0 Nasopharyngeal Carcinoma to Explore Different Treatment Modalities

**DOI:** 10.7150/jca.87901

**Published:** 2023-10-09

**Authors:** Chang Yan, Rong Zhao, Kai-Hua Chen, Biao-You Chen, Chao-Jun Zhang, Xi Chen, Wan-Wan Meng, Lin Lai, Song Qu, Xiao-Dong Zhu

**Affiliations:** 1 Department of Radiation Oncology, Guangxi Medical University Cancer Hospital, Nanning, Guangxi, 530021, People's Republic of China.; 2 Department of Oncology, Wuming Hospital of Guangxi Medical University, Nanning, Guangxi, 530199, People's Republic of China.

**Keywords:** nasopharyngeal carcinoma, survival, intensity-modulated radiotherapy, nomogram

## Abstract

**Purpose** To explore the prognostic value of clinical and serological risk factors for progression-free survival (PFS) in stage II and T3N0 nasopharyngeal carcinoma (NPC) and construct a nomogram based on these factors. Additionally, to investigate the long-term survival and short-term toxic reactions of patients in different risk stratification under different treatment modalities.

**Methods** The patients were randomly divided into training and validation cohorts in a 7:3 ratio. Independent prognostic factors were identified using Cox regression analysis, and a nomogram was constructed by combining these predictive factors with the TNM staging system. The nomogram was then validated in the validation cohort, and patients were classified into different risk groups based on the nomogram. The PFS, overall survival (OS), and acute toxicities were compared among different treatment modalities after balancing baseline characteristics.

**Results** Multivariate Cox regression analysis indicated that pathological type, alkaline phosphatase (ALP) and lactate dehydrogenase (LDH) were independent prognostic factors(p<0.05) in this study. The nomogram showed good prognostic accuracy in both the training and validation cohorts (C-index of 0.73 and 0.70, respectively). In the different risk subgroups, there were no statistically significant differences in PFS and OS between radiotherapy and chemoradiotherapy groups(p>0.05). The treatment modality of combined chemotherapy was associated with more acute toxic reactions.

**Conclusion** We established and validated a nomogram for predicting PFS in patients with stage II/T3N0 NPC. Intensity-modulated radiation therapy (IMRT) combined with chemotherapy did not provide additional survival benefits for these patients and was associated with more chemotherapy-related side effects.

## Introduction

Nasopharyngeal carcinoma (NPC) is a unique head and neck tumor that commonly occurs in southeastern China^1^, ^2^. Currently, the tumor, node, metastasis (TNM) staging system is one of the main criteria used by clinicians to determine treatment plans. Due to its specific anatomical location and biological characteristics, radiotherapy is the primary treatment method for NPC. With the development of intensity-modulated radiation therapy (IMRT), excellent treatment outcomes have been achieved with radiotherapy alone for early-stage NPC. However, there is still controversy surrounding the treatment modalities of patients with stage II and T3N0 NPC.

Adding chemotherapy to radiotherapy can enhance the local efficacy of radiation and eliminate micro-metastases^3^. However, platinum-based chemotherapy can increase acute toxic reactions during treatment, leading to treatment interruptions, weight loss, and decreased compliance, which may compromise treatment benefits^4^-^6^. In the era of two-dimensional (2D) radiotherapy, the standard treatment modality for patients with stage II and T3N0 NPC was concurrent chemoradiotherapy (CCRT) 7, 8. The National Comprehensive Cancer Network (NCCN) guidelines (2022, 2nd edition) and the Chinese Society of Clinical Oncology (CSCO) diagnosis and treatment guidelines (2022) recommend using IMRT alone as the treatment modality for stage T2N0 patients, while other stage II patients and stage T3N0 patients are advised to undergo CCRT. Additionally, induction chemotherapy (IC) combined with CCRT and CCRT with adjuvant chemotherapy (AC) are both considered as Class II recommendations for T3N0 patients according to the CSCO guidelines. However, a multicenter phase III clinical trial evaluated patients with stage II and T3N0 NPC without adverse features and found that the 3-year failure-free survival rate with IMRT alone was not inferior to that with concurrent chemoradiotherapy, and had fewer adverse reactions during treatment^9^. In the same way, a retrospective propensity-matched cohort study suggested that adding chemotherapy concurrently to IMRT did not significantly improve survival rates for patients with stage II and T3N0 disease and resulted in more severe toxic reactions 10.

Despite the generally favorable prognosis of patients with stage II and T3N0 NPC, a small proportion of patients still experience treatment failure 11. A reliable clinical prognostic model holds the potential to provide individualized treatment recommendations for patients with unfavorable prognoses. Currently, there is no prognostic model specifically designed for stage II and T3N0 NPC. In this study, we conducted prognostic analysis and developed a nomogram to differentiate patients' prognostic risks in this subset. Subgroup analysis further explored the long-term survival and toxicity reactions of patients with stage II and T3N0 NPC under different treatment modalities.

## Materials and Methods

### Study Population

A total of 434 patients diagnosed with stage II or T3N0 NPC at Guangxi Medical University Cancer Hospital between 2011 and 2017 were included. Inclusion criteria were as follows: ① Patients pathologically confirmed nasopharyngeal carcinoma; ② Retrospective staging as stage II or T3N0 according to the 8th edition of the American Joint Committee on Cancer (AJCC) staging system; ③ General good condition (KPS ≥ 70), without severe concurrent medical or surgical diseases, and no concurrent malignancies; ④ Complete clinical data available; ⑤ Treated by IMRT with or without chemotherapy.

### Data Collection and Follow-up

Baseline data collected included clinical information and serum parameters of all patients before treatment, such as gender, age, smoking history, pathological type, TNM staging, hemoglobin (HGB), albumin (ALB), alkaline phosphatase (ALP), lactate dehydrogenase (LDH), neutrophil-to-lymphocyte ratio (NLR), platelet-to-lymphocyte ratio (PLR), lymphocyte-to-monocyte ratio(LMR), and treatment modalities. The primary endpoint was progression-free survival (PFS), and secondary endpoints was overall survival (OS).

Follow-up was conducted through various means, including phone calls, outpatient visits, and hospital reexaminations. Within the first 2 years after treatment, patients were followed up every 3 months. In the third year, follow-ups were conducted every 6 months, and thereafter, annually. For the local and regional recurrence of NPC, our follow-up measures included symptoms inquiries, physical examinations, nasopharyngoscopy, MRI of the nasopharynx and neck, and testing for EBV DNA. Regarding distant metastasis, our important follow-up methods consisted of whole-body CT, PET/CT, bone scintigraphy, and EBV DNA.

### Treatment Methods

All patients received complete IMRT treatment with or without chemotherapy. Delineation of the target area and organs at risk followed the guidelines of International Commission on Radiation Units and Measurements Reports (ICRU) 50 and 62. The prescribed doses for target areas were as follows: GTVnx 70.06-75 Gy(30-32 fractions), GTVnd 60-73.6 Gy(30-32 fractions), CTV1 60-64 Gy(30-32 fractions), CTV2 54-57.6 Gy(30-32 fractions). Chemotherapy regimens included TP (docetaxel 75 mg/m2, day 1; cisplatin 75 mg/m2, day 1), PF (cisplatin 80 mg/m2, days 1-3; fluorouracil 750 mg/m2 continuous intravenous infusion over 120 hours), and TPF (docetaxel 60 mg/m2, day 1; cisplatin 60 mg/m2, day 1; fluorouracil 600 mg/m2 continuous intravenous infusion over 120 hours), administered every 3 weeks for 1-4 cycles for induction therapy. Concurrent chemotherapy consisted of cisplatin (100 mg/m2, days 1-3) every 3 weeks for 1-3 cycles. Adjuvant chemotherapy was administered with TPF, TP, or PF regimens, every 3 weeks for 1-4 cycles.

### Statistical Analysis

X-tile software (Chicago, Rim Lab) was employed to determine the optimal cut-off values for continuous variables of laboratory data, and they were subsequently divided into binary variables. Statistical analysis was conducted using the 25^th^ edition of SPSS for Windows (SPSS, Chicago, IL) software. Chi-square test or Fisher's exact test was performed to compare baseline characteristics and toxic reactions among different treatment groups. Patients were randomly assigned to training and validation cohorts in a 7:3 ratio. In the training cohort, univariate and multivariate Cox regression were used to identify independent prognostic factors for PFS. Kaplan-Meier method was performed for survival analysis, and survival rates were compared using the log-rank test. Independent prognostic factors were incorporated into the nomogram prediction model using R software version 4.3.0 (R project, http://www.R-project.org/). The concordance index (C-index) was calculated, and calibration curves were plotted to assess the accuracy of the model. X-tile software was used for risk stratification in the nomogram. Propensity score matching (PSM) with a caliper of 0.05 was employed in subgroup analysis to reduce selection bias between the radiotherapy and chemoradiotherapy groups. *P*-values of <0.05 were considered statistically significant for differences.

## Results

### Patient Characteristics

According to the inclusion criteria, we included 434 cases in this study. Among them, 304 patients were assigned to the training cohort, and 130 cases were assigned to the validation cohort. The baseline characteristics of the two cohorts are presented in Table 1. There were no statistically significant differences in baseline characteristics between the training and validation cohorts (p>0.05).

### Establishment and Validation of the Nomogram

**Figure 1:**
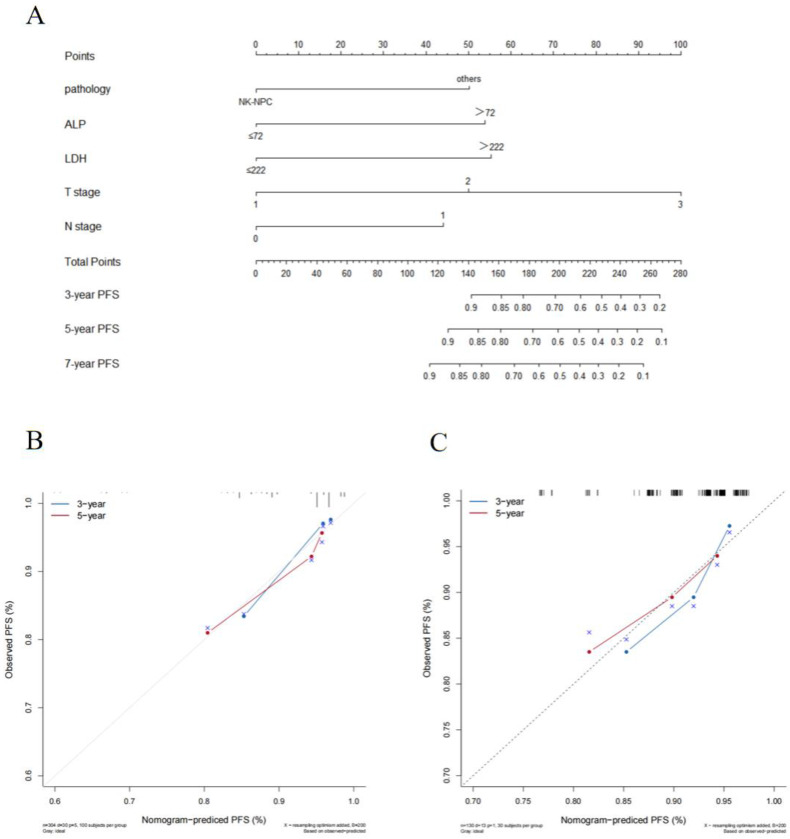
(A) Prognostic nomogram predicting NPC patients with stage II /T3N0 for PFS in the training cohort.The calibration curves of the nomogram for (B) the training cohort and (C) the validation cohort. Note: NK-NPC: non-keratinizing squamous cell carcinoma; ALP: alkaline phosphatase; LDH: lactate dehydrogenase; PFS: progression-free survival.

The cut-off values for HGB (137g/L), ALB (45.1g/L), ALP (72U/L), LDH (222U/L), NLR (2.2), PLR (114.4) and LMR (4.2) were calculated by X-tile software. In the training cohort, we explored the prognostic factors for PFS and constructed a nomogram. Univariate Cox regression analysis indicated that pathology, LDH, and ALP were significant prognostic factors for patients (p < 0.05). The factors with statistical significance in the univariate analysis were then incorporated into the multivariate Cox regression model, revealing that pathology, LDH, and ALP were independent prognostic factors (p < 0.05), as shown in Table 2. Although T stage and N stage were not independent prognostic factors in the Cox regression analysis of this study, their importance is widely recognized 12. Therefore, we constructed the nomogram for PFS by combining the independent prognostic factors with TNM stage (Figure 1A).

The C-index of the nomogram was 0.73(95% CI: 0.63-0.83) in the training cohort and 0.70(95% CI: 0.54-0.86) in the validation cohort which shows good distinction. The calibration curves in both cohorts closely approximated the standard curve, indicating high accuracy of the nomogram (Figure 1B and C).

### Risk Stratification and Survival Analysis

**Figure 2:**
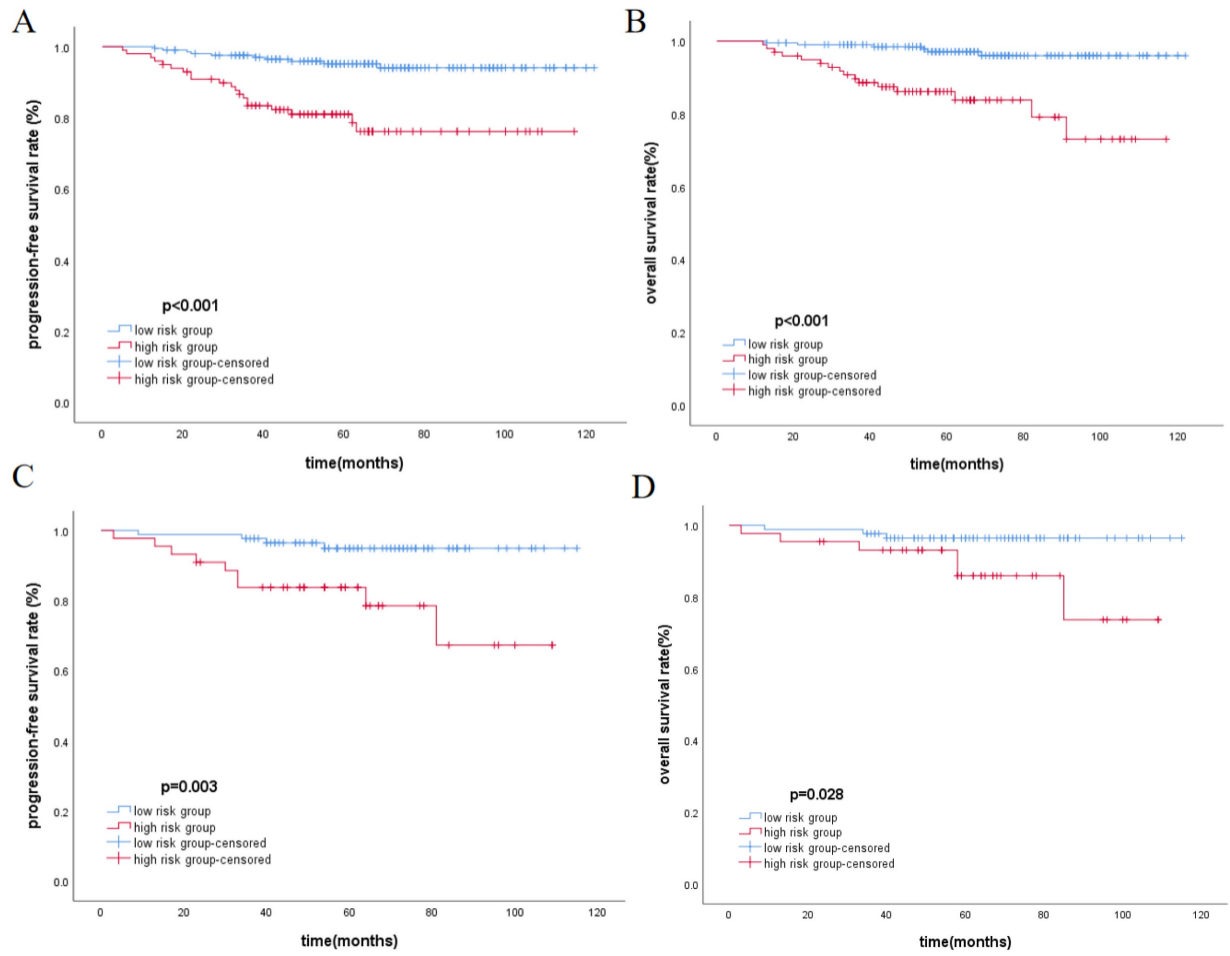
Kaplan-Meier analysis for PFS and OS in training cohort(A, B) and validation cohort(C, D), respectively.

The total score of the nomogram was calculated for each patient, and the X-tile software was used to select the optimal cut-off value of 104 points. Based on this value, patients were divided into the low-risk group (≤104) and the high-risk group (>104). The Kaplan-Meier method was used to plot survival curves, indicating that in the training cohort and validation cohort, the low-risk group had significantly better PFS and OS compared to the high-risk group (p<0.05), as shown in Figure 2. In the overall cohort, the 5-year PFS for the low-risk group was 95.1%, while it was 81.9% for the high-risk group. Similarly, patients in the low-risk group achieved better OS, with 5-year OS rates of 96.9% for the low-risk group and 85.9% for the high-risk group.

### Toxicity and Subgroup Survival Analysis

**Figure 3:**
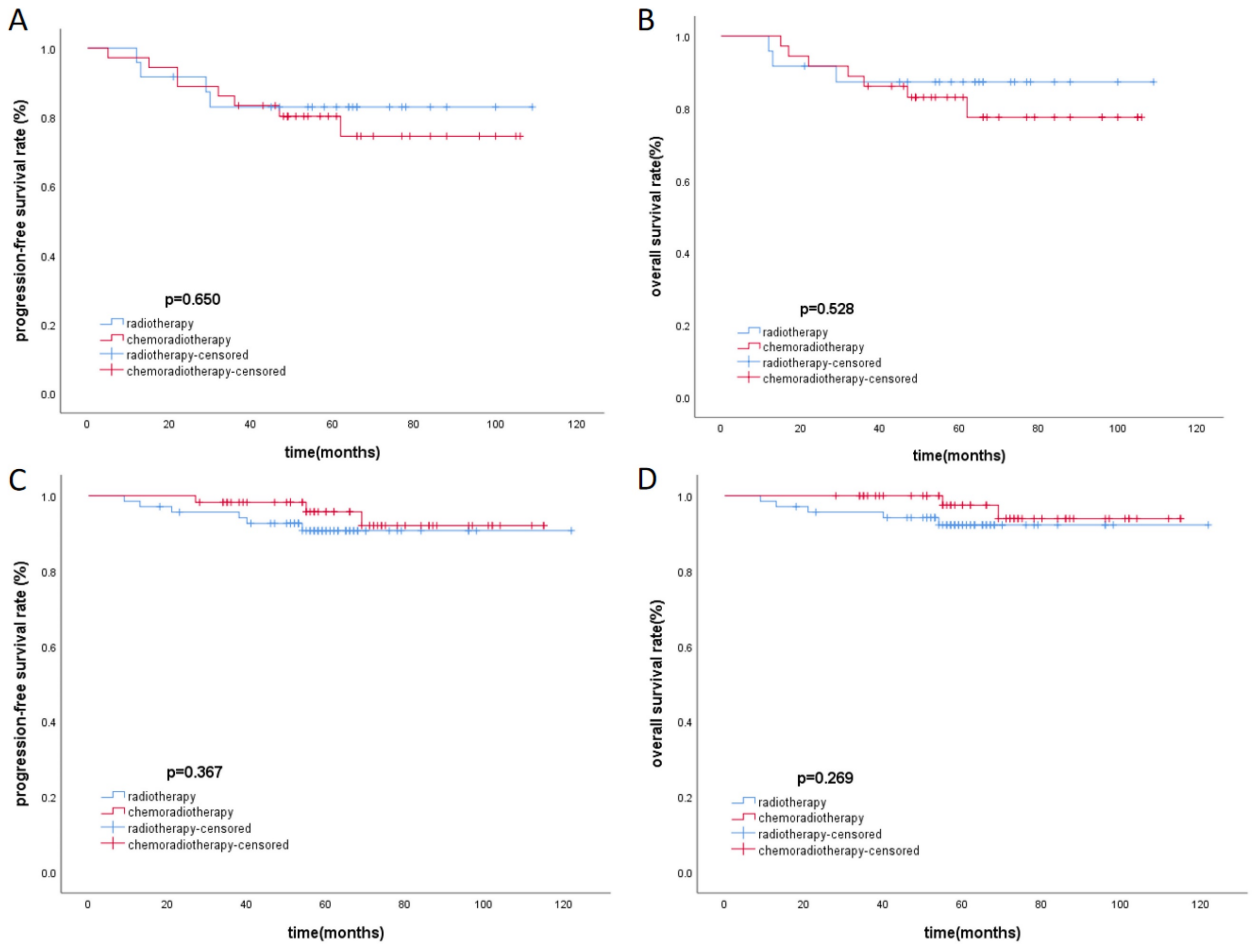
Kaplan-Meier survival curves of PFS and OS in high-risk (A, B) group and low-risk group (C, D) respectively.

There was baseline data imbalance between the radiotherapy group and the chemoradiotherapy group. After propensity score matching (with a strict caliper of 0.05), 93 pairs of cases were selected, which balanced the baseline differences between the two cohorts (Table 3). Acute toxicities during treatment were evaluated in the PSM cohort, including the radiotherapy group and the chemoradiotherapy group. Compared to the radiotherapy group, patients in the chemoradiotherapy group had higher rates of grade 1-4 leukopenia, anemia, thrombocytopenia, and gastrointestinal reactions (p<0.05). Moreover, the chemoradiotherapy group had a higher proportion of patients experiencing severe grade 3-4 leukopenia (Table 4). In the low-risk group and the high-risk group after PSM, Kaplan-Meier survival analysis showed no statistically significant differences in PFS and OS under different treatment methods (Figure 3).

## Discussion

This study explored the clinical independent prognostic factors for stage II and T3N0 NPC and successfully constructed a nomogram incorporating the TNM staging system. The nomogram we developed successfully stratified patients with stage II and T3N0 NPC into high-risk and low-risk groups. However, in different risk groups, the addition of chemotherapy to radiotherapy did not improve patients' PFS and OS and increased more chemotherapy-related toxicities.

The TNM staging system is currently the most reliable staging system for NPC and is commonly used in clinical practice to assist doctors in making treatment decisions^13^. However, there is still controversy regarding the treatment approach for patients with stage II and T3N0 NPC. These patients have similar T and N stages, making it difficult to distinguish patients with different prognoses. A reliable prognostic model is expected to provide a basis for individualized treatment strategies in clinical practice.

We explored the independent prognostic factors for PFS in this staging, including pathological type, ALP, and LDH. Previously, researchers believed that adverse prognostic factors for patients with stage II and T3N0 included extracapsular extension of cervical lymph nodes^14^, lymph node cross-sectional diameter ≥3 cm, positive lymph nodes in the IV/Vb region^15^, and pre-treatment plasma EB viral DNA copy number ≥4000 copies/ml^16^. Our study is expected to provide additional insights into adverse prognostic factors for patients in this staging of nasopharyngeal carcinoma. According to the 5th edition of the WHO classification of head and neck tumors, NPC can be classified into three subtypes:(i) non-keratinizing squamous cell carcinoma (NK-NPC), (ii) keratinizing squamous cell carcinoma(K-NPC), and (iii) basaloid squamous cell carcinoma (basaloid SCC)^17^. The histological subtypes of nasopharyngeal carcinoma exhibit significant geographical and racial distribution^18^. In our study, NK-NPC patients had a better prognosis, which is consistent with previous research findings^19^, ^20^. Although undifferentiated tumors are generally considered more invasive, NK-NPC has been shown to have higher radiosensitivity, which may be a contributing factor to the different prognosis^21^.

LDH is an enzyme in the glycolytic pathway, and its levels increase with the release of anaerobic metabolism in malignant tumors^22^. Elevated LDH has been widely reported to indicate poor prognosis in various types of tumors^23^. The reason for the association between high LDH levels and poor tumor prognosis may be related to the hypoxic environment associated with high tumor burden, leading to increased LDH production^24^. Additionally, elevated LDH levels may lead to upregulation of the HIF pathway, increased expression of vascular endothelial growth factor, and weakened immune function^25^-^27^, all of which could contribute to adverse tumor outcomes. In our study, LDH>222U/L was identified as an independent risk factor for disease progression, which aligns with these previous research findings. ALP is a phosphomonoesterase related to human bone metabolism^28^. Although elevated ALP has been shown to be a poor prognostic factor in various tumors^29^-^31^, the exact reasons for its impact on NPC prognosis remain unclear. A study found that elevated ALP levels were common in patients with T3-T4 stage NPC and speculated a connection with skull base invasion in these patients^32^. In our study, we found that elevated ALP levels were also associated with poor prognosis in stage II and T3N0 patients, suggesting that ALP might be involved in other mechanisms contributing to adverse NPC prognosis. Previous research has shown that elevated ALP is closely related to lymph node involvement^33^. A fundamental study also suggested that ALP might promote proliferation at the cellular level^34^. Furthermore, high levels of ALP have been linked to occult metastasis, which cannot be detected by imaging^35^. Therefore, we believe that elevated ALP levels can be used for risk assessment of poor prognosis in stage II and T3N0 patients.

While HGB, ALB, NLR, and other indicators in this study have been shown to be associated with NPC prognosis^36^-^38^, they were not independent prognostic factors in this study. This may be attributed to the differences in tumor staging and cutoff value selection in different studies. T and N staging, although representing important prognostic indicators, were not independent prognostic factors in this study, possibly due to the early staging and similar staging of this subgroup of patients. Considering that TNM staging is one of the most reliable prognostic indicators in clinical practice, we included it along with other independent prognostic factors in our nomogram. Our prognostic model incorporates commonly available clinical and serological parameters that are easily obtained and analyzed in clinical practice.

Through the nomogram, we divided patients with stage II and T3N0 NPC into high and low-risk subgroups, showing significant differences in PFS and OS (p<0.05). In subgroup survival analysis, the addition of chemotherapy did not provide additional survival benefits to different risk groups or the overall population. On the contrary, the use of chemotherapy increased the occurrence of more acute toxic reactions and severe grade 3-4 acute reactions. Recently, immunotherapy drugs such as pembrolizumab and camrelizumab have made breakthrough progress in the treatment of recurrent or metastatic NPC^39^, ^40^. Additionally, a large-scale retrospective study with long-term follow-up suggested that the addition of cetuximab and nimotuzumab to chemoradiotherapy may effectively maximize the survival of patients with stage II-IVb nasopharyngeal carcinoma^41^. Whether high-risk patients in stage II and T3N0 can benefit from immunotherapy or targeted therapy, or whether optimizing low-toxicity chemotherapy regimens, is worth considering and exploring in clinical trials. Our study provides evidence for evidence-based medicine in this regard. Compared with the TNM staging system, the nomogram we built contains a border type of clinical and characteristics, which could reflect the biological differences of different patients. The study is expected to provide a basis for clinicians to develop individualized treatment strategies for NPC patients in stage II and T3N0.

This study has certain limitations. Firstly, it is a single-center retrospective study, which may introduce selection bias in patient selection. Secondly, EBV-DNA, as a potential prognostic factor^42^, was not included in our study. This is because EBV-DNA was not a routine examination in our center, and there was currently no unified method for detecting EBV-DNA among different laboratories, leading to significant variations in results. Thirdly, this study is retrospective, and we did not completely standardize the diagnostic and treatment approaches for different patients, which may have influenced the results to some extent. Furthermore, we did not stratify the different chemotherapy methods, primarily due to the limited sample size in this staging group. These limitations need to be addressed in future studies.

## Conclusion

We explored the independent prognostic factors of stage II and T3N0 NPC and validated them by constructing a nomogram. The nomogram successfully classified patients in this stage into high-risk and low-risk groups. The addition of chemotherapy to radiotherapy did not provide survival benefits in different risk groups and led to more chemotherapy-related adverse reactions. Further prospective exploration of treatment strategies for high-risk patients in this stage is needed.

## Figures and Tables

**Table 1:** Baseline characteristics of patients with stage II/T3N0 NPC in training cohort and validation cohort

Clinical factors		Total cohort	Training cohort	Validation cohort	*p*-value
		(N = 434)	(N =304)	(N = 130)	
Age(years)	≤50	284(65.4%)	199(65.5%)	85(65.4%)	0.988
	>50	150(34.6%)	105(34.5%)	45(34.6%)	
Gender	male	315(72.6%)	225(74.0%)	90(69.2%)	0.306
	female	119(27.4%)	79(26.0%)	40(30.8%)	
Smoking	No	301(69.3%)	215(70.7%)	86(65.9%)	0.319
	Yes	133(30.7%)	89(29.3%)	44(34.1%)	
pathology	K-NPC/ basaloid SCC	46(10.6%)	33(10.9%)	13(10.0%)	0.791
	NK-NPC	388(89.4%)	271(89.1%)	117(90.0%)	
T stage	T1	58(13.4%)	39(12.8%)	19(14.6%)	0.359
	T2	357(82.2%)	249(81.9%)	108(83.1%)	
	T3	19(4.4%)	16(5.3%)	3(2.3%)	
N stage	N0	68(15.7%)	48(15.8%)	20(15.4%)	0.915
	N1	366(84.3%)	256(84.2%)	110(84.6%)	
Clinical stage	C2	415(95.6%)	288(94.7%)	127(97.7%)	0.168
	C3	19(4.4%)	16(5.3%)	3(2.3%)	
Treatment	RT	98(22.6%)	71(23.4%)	27(20.8%)	0.605
	CCRT	262(60.4%)	181(59.5%)	81(62.3%)	
	IC+CCRT	26(6.0%)	16(5.3%)	10(7.7%)	
	CCRT+AC	48(11.1%)	36(11.8%)	12(9.2%)	
HGB(g/L)	≤137	169(38.9%)	119(39.1%)	50(38.5%)	0.894
	>137	265(61.1%)	185(60.9%)	80(61.5%)	
ALB(g/L)	≤45.1	283(65.2%)	198(65.1%)	85(65.4%)	0.960
	>45.1	151(34.8%)	106(34.9%)	45(34.6%)	
ALP(U/L)	≤72	319(73.5%)	222(73.0%)	97(74.6%)	0.731
	>72	115(26.5%)	82(27.0%)	33(25.4%)	
LDH(U/L)	≤222	386(88.9%)	276(90.8%)	110(84.6%)	0.060
	>222	48(11.1%)	28(9.2%)	20(15.4%)	
NLR	≤2.2	261(60.1%)	189(62.2%)	72(55.4%)	0.186
	>2.2	173(39.9%)	115(37.8%)	58(44.6%)	
PLR	≤114.4	150(34.6%)	106(34.9%)	44(34.1%)	0.837
	>114.4	284(65.4%)	198(65.1%)	86(65.9%)	
LMR	≤4.2	142(32.7%)	100(32.9%)	42(32.3%	0.905
	>4.2	292(67.3%)	204(67.1%)	88(67.7%)	
Progression	No	391(90.1%)	274(90.1%)	117(90.0%)	0.966
	Yes	43(9.9%)	30(9.9%)	13(10.0%)	

Note: NK-NPC: non-keratinizing squamous cell carcinoma; K-NPC: keratinizing squamous cell carcinoma; basaloid SCC: basaloid squamous cell carcinoma; RT: radiotherapy; CCRT: concurrent chemoradiotherapy; IC: induction chemotherapy; AC: adjuvant chemotherapy; HGB: hemoglobin; ALB: albumin; ALP: alkaline phosphatase; LDH: lactate dehydrogenase; NLR: neutrophil-to-lymphocyte ratio; PLR: platelet-to-lymphocyte ratio; LMR: Lymphocyte-monocyte ratio.

**Table 2:** Univariate and multivariable cox analysis of the risk fators for PFS in the training cohort

Characteristic	Univariate Cox regression analysis		Multivariate Cox regression analysis	
	HR (95% CI)	*p*-value	HR (95% CI)	*p-*value
Gender (Female, Male)	0.900(0.386~2.098)	0.807	-	-
Age (Years; ≤50, >50)	1.737(0.848~3.559)	0.131	-	-
Smoking (No, Yes)	0.838(0.373~1.884)	0.670	-	-
pathology (Others, NK-NPC)	0.376(0.161~0.877)	0.024	0.324(0.138~0.759)	0.009
Treatment (radiotherapy, chemoradiotherapy)	1.332(0.764~2.324)	0.312	-	-
HGB (g/L; ≤137, >137)	0.709(0.346~1.454)	0.348	-	-
ALB (g/L; ≤45.1, >45.1)	0.683(0.311~1.501)	0.342	-	-
ALP (U/L; ≤72, >72)	3.130(1.523~6.432)	0.002	3.181(1.532~6.604)	0.002
LDH (U/L; ≤222, >222)	3.966(1.765~8.913)	<0.001	3.559(1.570~8.066)	0.002
NLR (≤2.2, >2.2)	1.741(0.851~3.562)	0.129	-	-
PLR (≤114.4, >114.4)	1.859(0.798~4.333)	0.151	-	-
LMR (≤4.2, >4.2)	0.522(0.255~1.071)	0.076	-	-
T stage (T1, T2-3)	2.056(0.490~8.631)	0.325	-	-
N stage (N0, N1)	1.729(0.525~5.701)	0.368	-	-
Clinical stage (II, III)	1.472(0.350~6.184)	0.598	-	-

Note: Female, age≤50 years old, no smoking history, other pathology, radiotherpy, HGB≤137g/L, ALB≤45.1g/L, ALP≤72U/L, LDH≤222U/L, NLR≤2.2, PLR≤114.4, LMR≤4.2, T1, N0 and clinical stage of II were the reference groups. NK-NPC: non-keratinizing Squamous Cell Carcinoma; HGB: hemoglobin; ALB: albumin; ALP: alkaline phosphatase; LDH: lactate dehydrogenase; NLR: neutrophil-to-lymphocyte ratio; PLR: platelet-to-lymphocyte ratio; LMR: Lymphocyte-monocyte ratio.

**Table 3:** Patients characteristics in unmatched cohort and matched cohort

Clinical Factors		Before Matching			After Matching		
		Radiotherapy	Chemoradiotherapy	*p*-value	Radiotherapy	Chemoradiotherapy	*p*-value
		(N=98)	(N=336)		(N=93)	(N=93)	
Age(years)	≤50	51(52.0%)	233(69.3%)	0.002	50(53.8%)	53(57.0%)	0.658
	>50	47(48.0%)	103(30.7%)		43(46.2%)	40(43.0%)	
Gender	Male	70(71.4%)	245(72.9%)	0.771	66(71.0%)	72(77.4%)	0.315
	Female	28(28.6%)	91(27.1%)		27(29.0%)	21(22.6%)	
Smoking	No	69(70.4%)	232(69.0%)	0.797	64(68.8%)	53(57.0%)	0.095
	Yes	29(29.6%)	104(31.0%)		29(31.2%)	40(43.0%)	
pathology	K-NPC/basaloid SCC	12(12.2%)	34(10.1%)	0.547	11(11.8%)	12(12.9%)	0.824
	NK-NPC	86(87.8%)	302(89.9%)		82(88.2%)	81(87.1%)	
T stage	T1	15(15.3%)	43(12.8%)	0.521	14(15.1%)	9(9.7%)	0.265
	T2-3	83(84.7%)	293(87.2%)		79(84.9%)	84(90.3%)	
N stage	N0	23(23.%)	45(13.4%)	0.016	18(19.4%)	20(21.5%)	0.716
	N1	75(76.5%)	291(86.6%)		75(80.6%)	73(78.5%)	
HGB(g/L)	≤137	45(45.9%)	124(36.9%)	0.107	41(44.1%)	42(45.2%)	0.883
	>137	53(54.1%)	212(63.1%)		52(55.9%)	51(54.8%)	
ALB(g/L)	≤45.1	69(70.4%)	214(63.7%)	0.219	65(69.9%)	66(71.0%)	0.872
	>45.1	29(29.6%)	122(36.3%)		28(30.1%)	27(29.0%)	
ALP(U/L)	≤72	77(78.6%)	242(72.0%)	0.196	74(79.6%)	66(71.0%)	0.174
	>72	21(21.4%)	94(28.0%)		19(20.4%)	27(29.0%)	
LDH(U/L)	≤222	87(88.8%)	299(89.0%)	0.935	84(90.3%)	81(87.1%)	0.487
	>222	11(11.2%)	37(11.0%)		9(9.7%)	12(12.9%)	
NLR	≤2.2	67(68.4%)	261(60.1%)	0.059	63(67.7%)	52(55.9%)	0.097
	>2.2	31(31.6%)	173(39.9%)		30(32.3%)	41(44.1%)	
PLR	≤114.4	33(33.7%)	117(34.8%)	0.833	32(34.4%)	30(32.3%)	0.756
	>114.4	65(66.3%)	219(65.2%)		61(65.6%)	63(67.7%)	
LMR	≤4.2	30(30.6%)	112(33.3%)	0.613	29(31.2%)	34(36.6%)	0.439
	>4.2	68(69.4%)	224(66.7%)		64(68.8%)	59(63.4%)	

Note: K-NPC: keratinizing squamous cell carcinoma; basaloid SCC: basaloid squamous cell carcinoma; NK-NPC: non-keratinizing squamous cell carcinoma; HGB: hemoglobin; ALB: albumin; ALP: alkaline phosphatase; LDH: lactate dehydrogenase; NLR: neutrophil-to-lymphocyte ratio; PLR: platelet-to-lymphocyte ratio; LMR: Lymphocyte-monocyte ratio.

**Table 4:** Acute toxic reactions of patients in the radiotherapy group and chemoradiotherapy group

Adverse Event		Radiotherapy	Chemoradiotherapy	*p*-value
		(N=93)	(N=93)	
Leucopenia	All	46(49.5%)	77(82.8%)	<0.001
	Grade 3-4	0(0.0%)	23(24.7%)	<0.001
Anemia	All	0(0.0%)	31(33.3%)	<0.001
	Grade 3-4	0(0.0%)	2(2.2%)	0.497
Thrombocytopenia	All	0(0.0%)	8(8.6%)	0.007
	Grade 3-4	0(0.0%)	1(1.1%)	1.000
Liver Dysfunction	All	11(11.8%)	3(3.2%)	0.050
	Grade 3-4	0(0.0%)	0(0.0%)	-
Gastrointestinal reaction	All	8(8.6%)	52(55.9%)	<0.001
	Grade 3-4	0(0.0%)	5(5.4%)	0.059

## Abbreviations

NPC: nasopharyngeal carcinoma
PFS: progression-free survival
OS: overall survival
ALP: alkaline phosphatase
LDH: lactate dehydrogenase
IMRT: intensity-modulated radiation therapy
NCCN: The National Comprehensive Cancer Network
AJCC staging system: the American Joint Committee on Cancer staging system
CSCO: the Chinese Society of Clinical Oncology
CCRT: concurrent chemoradiotherapy
IC: induction chemotherapy
AC: adjuvant chemotherapy
ICRU: International Commission on Radiation Units and Measurements Reports
C-index: The concordance index
PSM: Propensity score matching
HGB: hemoglobin
ALB: albumin
NLR: neutrophil-to-lymphocyte ratio
PLR: platelet-to-lymphocyte ratio
LMR: Lymphocyte-monocyte ratio
NK-NPC: non-keratinizing Squamous Cell Carcinoma
K-NPC: keratinizing Squamous Cell Carcinoma
basaloid SCC: basaloid Squamous Cell Carcinoma
